# Neutrophil P2X_7_ receptors mediate NLRP3 inflammasome-dependent IL-1β secretion in response to ATP

**DOI:** 10.1038/ncomms10555

**Published:** 2016-02-15

**Authors:** Mausita Karmakar, Michael A. Katsnelson, George R. Dubyak, Eric Pearlman

**Affiliations:** 1 Department of Physiology and Biophysics, Case Western Reserve University, Cleveland, Ohio 44106, USA; 2 Department of Ophthalmology and Visual Sciences, Case Western Reserve University, Cleveland, Ohio 44106, USA; 3 Department of Physiology and Biophysics and Department of Ophthalmology, University of California, Irvine, California 92697, USA

## Abstract

Although extracellular ATP is abundant at sites of inflammation, its role in activating inflammasome signalling in neutrophils is not well characterized. In the current study, we demonstrate that human and murine neutrophils express functional cell-surface P2X_7_R, which leads to ATP-induced loss of intracellular K^+^, NLRP3 inflammasome activation and IL-1β secretion. ATP-induced P2X_7_R activation caused a sustained increase in intracellular [Ca^2+^], which is indicative of P2X_7_R channel opening. Although there are multiple polymorphic variants of P2X_7_R, we found that neutrophils from multiple donors express P2X_7_R, but with differential efficacies in ATP-induced increase in cytosolic [Ca^2+^]. Neutrophils were also the predominant P2X_7_R-expressing cells during *Streptococcus pneumoniae* corneal infection, and P2X_7_R was required for bacterial clearance. Given the ubiquitous presence of neutrophils and extracellular ATP in multiple inflammatory conditions, ATP-induced P2X_7_R activation and IL-1β secretion by neutrophils likely has a significant, wide ranging clinical impact.

## Abstract (web summary)

Neutrophils are a major source of IL-1 β in a number of inflammatory settings. Here the authors show that mouse and human neutrophils express functional P2X7 receptors, which mediate ATP-triggered NLRP3 inflammasome activation and IL-1 ß secretion.

## Graphical abstract

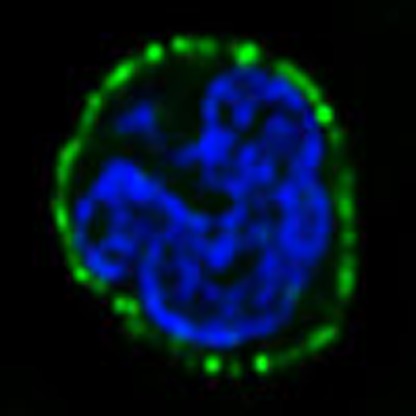

The inflammatory process results in tissue damage that can impair organ function, which can result in clinical manifestations. Almost all mammalian cells including myeloid cells, platelets, leukocytes, epithelial and endothelial cells can release ATP^1^, which can then lead to paracrine or autocrine activation of downstream purinergic signalling and exacerbation of the inflammatory response^2^. ATP activates plasma membrane purinergic receptors of the P2X and P2Y families that are expressed on many cell types, including myeloid and lymphoid cells^3^. Extracellular ATP has been implicated in multiple *in vivo* inflammatory responses, including lung inflammation and fibrosis, systemic inflammation and tissue damage during endotoxemia^4^,^5^,^6^.

The ionotropic P2X_7_ receptor (P2X_7_R) is expressed on macrophages and dendritic cells, which is activated by extracellular ATP to induce NLRP3 inflammasome assembly and caspase-1-dependent processing and release of the proinflammatory cytokines interleukin (IL)-1β and IL-18 (ref. 7). In addition to macrophages and dendritic cells, expression of functional P2X_7_R has been described in other human and murine hematopoietic lineage cells, including mast cells, B and T lymphocytes, monocytes, microglial cells and osteoclasts^8^; however, reports of P2X_7_R expression and its function on human and murine neutrophils are conflicting, and therefore the role of P2X_7_R in these cells remains uncertain.

P2X_7_R messenger RNA expression was reported in both human peripheral blood neutrophils and in the HL-60 human promyelocytic leukemia cells that were differentiated into a granulocyte lineage^9^,^10^. Another report showed the presence of P2X_7_R protein in the cytosol, but not on the cell surface of human neutrophils^11^, whereas other investigators did not detect P2X_7_R messenger RNA or protein in human neutrophils^11^,^12^,^13^,^14^. We are aware of only one report showing ATP-induced activation of inflammasome signalling in murine neutrophils^15^, although those investigators did not identify the ATP receptor. Notably, no systemic studies on P2X_7_R RNA or protein expression in murine neutrophils have been performed. Given that neutrophils are the predominant cell type in multiple causes of acute infection and inflammation, and that ATP is released at sites of inflammation, we addressed these apparent contradictions in the literature by using multiple approaches to characterize P2X_7_R expression and function in human peripheral blood neutrophils and in murine bone marrow neutrophils.

We now report that extracellular ATP triggers rapid increase in cytosolic Ca^2+^ and K^+^ efflux and robust IL-1β secretion in human and murine neutrophils via a P2X_7_R-regulated inflammasome platform that requires NLRP3, ASC and caspase-1. We further extended our analysis of human peripheral blood neutrophils by testing multiple healthy donors (*n*=11) from different ethnic backgrounds and confirmed cell-surface P2X_7_R expression as well as P2X_7_R-mediated increases in cytosolic Ca^2+^ and IL-1β secretion in response to ATP. Although the magnitude of the responses was variable, there was no association with donor ethnicity. Lastly, using a murine model of corneal infection, we demonstrated the *in vivo* relevance of these findings by showing that P2X_7_R-expressing neutrophils are recruited into *Streptococcus pneumoniae* infected corneas, and that P2X_7_R expression on neutrophils regulates bacterial survival in the tissues. Given the abundance of extracellular ATP generated during inflammatory processes, P2X_7_R activation on neutrophils may be a potential target to regulate tissue damage.

## Results

### ATP induces IL-1β secretion by C57BL/6 neutrophils

To examine IL-1β secretion by neutrophil in response to extracellular ATP, bone marrow neutrophils from C57BL/6 mice were primed with lipopolysaccharide (LPS), and stimulated with ATP in the presence or absence of apyrase, which catalyses rapid hydrolysis of ATP to ADP and AMP.

**Figure 1:**
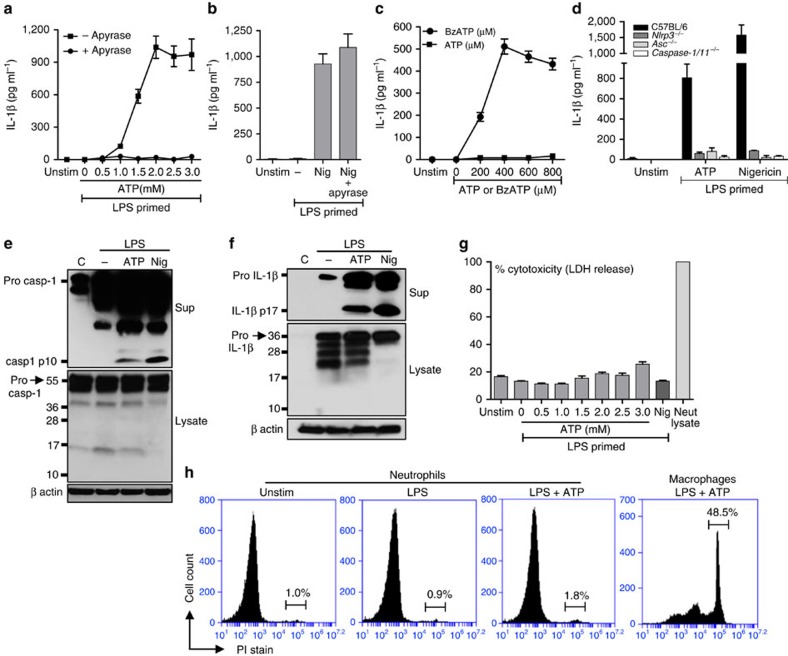
ATP—induced IL-1β secretion by murine bone marrow neutrophils. (**a**,**b**). IL-1β in the cell supernatants of C57BL/6 bone-marrow-derived neutrophils primed 3 h with LPS (500 ng ml^−1^) and stimulated 45 min with ATP (0.5–3.0 mM) or 10 μM nigericin. Apyrase (10U ml^−1^) was added 30 min before stimulation with ATP or nigericin, and IL-1β secretion was quantified by ELISA. (**c**). IL-1β production by LPS-primed C57BL/6 murine neutrophils stimulated 45 min with BzATP. (**d**). IL-1β in supernatants of LPS-primed bone-marrow-derived neutrophils from C57BL/6, *Nlrp3*^−/−^, *Asc*^−/−^ and *Caspase1,11*^−/−^ mice after 45 min stimulation with 3 mM ATP or 10 μM nigericin. (**e**,**f**). Pro- and cleaved forms of caspase-1 and IL-1β in total cell lysates and TCA precipitated supernatant of LPS-primed C57BL/6 neutrophils after 45 min stimulation with 3 mM ATP or 10 μM nigericin. (**g**). LDH release as a measure of cytotoxicity in LPS-primed C57BL/6 neutrophils after ATP stimulation. (**h**). Propidium iodide (PI) uptake by bone marrow neutrophils and bone-marrow-derived macrophages following LPS priming and 45 min ATP (3 mM) stimulation. Data were generated by flow cytometry. Data points are mean±s.d. of at least four replicates per treatment and are representative of three independent experiments.

As shown in Fig. 1a, ATP stimulated IL-1β secretion by LPS-primed neutrophils in a dose-dependent manner. Further, apyrase ablated ATP-induced IL-1β secretion, but had no effect on IL-1β secretion induced by the K^+^ ionophore nigericin (Fig. 1b). Similarly, neutrophils that were primed with heat killed *S. pneumoniae* (hkSP) secreted IL-1β following ATP stimulation, which was ablated in the presence of apyrase (Supplementary Fig. 1B,C). Notably, millimolar concentrations of ATP (effector concentration for half-maximum response (EC_50_)∼1.3 mM) were required for robust IL-1β release, which is consistent with the low affinity of P2X_7_R for ATP—a defining hallmark of P2X_7_R pharmacology^16^. In contrast, IL-1β production by LPS-primed neutrophils stimulated with 3-*O*-benzoylbenzoic acid-derivatized ATP analogue (BzATP) was maximal at 300 μM (Fig. 1c), consistent with the well-characterized higher affinity of P2X_7_R for BzATP^17^. IL-1β was not produced when neutrophils were stimulated with UTP, which targets the G-protein-coupled P2Y_2_ receptors that are highly expressed in murine and human neutrophils (Supplementary Fig. 1D).

To examine whether ATP-induced IL-1β secretion is dependent on the NLRP3/ASC inflammasome, bone marrow neutrophils from C57BL/6, *Nlrp3*^−/−^, *Asc*^−/−^ and *Caspase1/11*^−/−^ mice were primed with LPS, and stimulated with ATP or nigericin. As shown in Fig. 1d, IL-1β secretion induced by either ATP or nigericin was significantly lower in *Nlrp3*^−/−^, *Asc*^−/−^ and Caspase-1/11^−/−^ neutrophils compared with C57BL/6 cells, indicating dependence on this pathway. Further, neutrophils stimulated with ATP or nigericin rapidly secreted precursor and mature forms of caspase-1 and IL-1β, which was detected in the extracellular supernatants (Fig. 1e,f).

As caspase-1 mediates pyroptosis in macrophages and dendritic cells^18^, we examined whether ATP-stimulated neutrophils undergo pyroptotic cell death by measuring release of lactate dehydrogenase (LDH) and uptake of propidium iodide (PI). As shown in Fig. 1g, there was no increased LDH release in ATP-stimulated neutrophils compared with unstimulated neutrophils. Similarly,<2% of neutrophils stimulated with LPS plus ATP had intracellular PI, whereas 48.5% of bone-marrow-derived macrophages (BMDMs) were PI+ after 45 min (Fig. 1h).

Thus, the release of precursor and mature forms of both caspase-1 and IL-1β by ATP-stimulated neutrophils is mediated by the NLRP3 inflammasome and is regulated by a non-lytic mechanism rather than neutrophil pyroptosis.

### Murine neutrophils express functional P2X_7_R

Although P2X_7_R mediates ATP-induced IL-1β secretion by macrophages and dendritic cells^16^,^19^, the role of this receptor on murine neutrophils has yet to be characterized. The high concentration of extracellular ATP required for IL-1β secretion by murine neutrophils (Fig. 1) is consistent with P2X_7_R characteristics. To examine this hypothesis, neutrophils and BMDMs from C57BL/6 and *P2X*_*7*_^−/−^ mice were primed with LPS, and analysed for P2X_7_R expression using antibodies that recognize epitopes in either the intracellular C terminus domain (C-term antibody) or the extracellular domain (ecto antibody) of the receptor.

**Figure 2:**
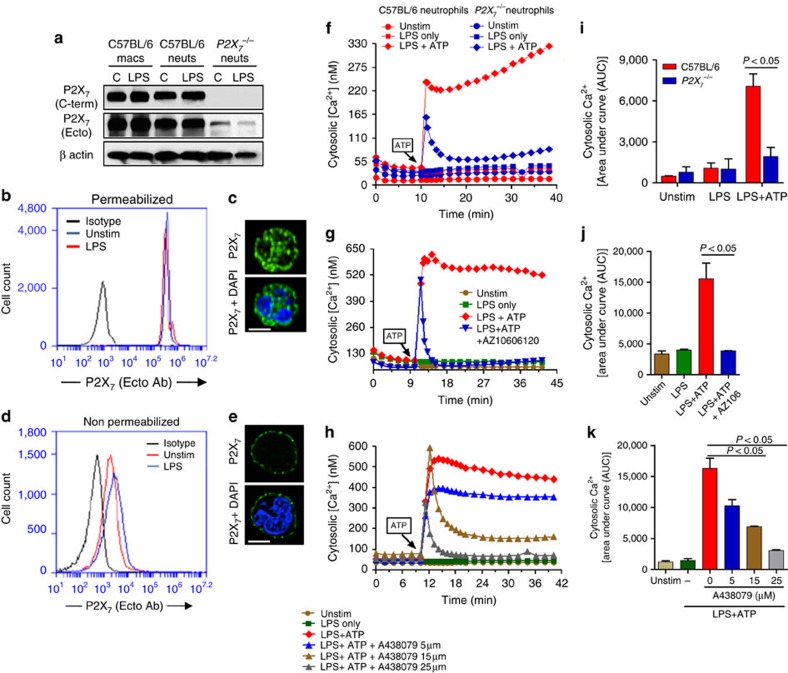
P2X_7_ receptor expression and function in murine bone marrow neutrophils. (**a**). Western blot analysis of P2X_7_ receptors on unstimulated and LPS-primed bone marrow neutrophils and macrophages from C57BL/6 and *P2X_7_*^−/−^ mice. (**b**–**e**). Flow cytometry and fluorescence microscopy of P2X_7_R ecto domain (using the Hano-3 antibody) in LPS-primed bone marrow neutrophils that were either permeabilized with 0.1% TX-100 to detect total P2X_7_R (**b**,**c**) or were non-permeabilized to detect only cell surface P2X_7_R (**d**,**e**). (**c,e**) Cells were also stained with DAPI to detect the nucleus; Scale bar, 5 μm). (**f**–**h**). Representative profiles of ATP-induced Ca^2+^ influx in bone marrow neutrophils from C57BL/6 and *P2X_7_*^−/−^ mice (**f**), and in C57BL/6 neutrophils after stimulation with ATP (3 mM) in the presence of P2X_7_R antagonists AZ10606120 (10 μM) (**g**), or with different concentrations of A438079 (**h**). (**i**–**k**). Mean±s.e.m. pf Area under the curve (AUC) of intracellular Ca^2+^ from three independent experiments. A *P* value ≤0.05 was considered significant using an unpaired Student's *t-*test.

BMDMs from C57BL/6 mice exhibited robust constitutive expression P2X_7_R (Fig. 2a), as reported previously^16^,^19^. Further, C57BL/6 neutrophils constitutively expressed P2X_7_R as detected by western blot analysis (Fig. 2a). In contrast, *P2X*_*7*_^−/−^ neutrophil cell lysate did not react with the C terminus antibody, but showed low-level immunoreactivity with the ecto-domain antibody indicating the presence of truncated C-terminal receptor variants described for the Pfizer *P2X*_*7*_^−/−^ mice^20^.

To discriminate between cell surface and intracellular P2X_7_R protein, LPS-primed C57BL/6 neutrophils were permeabilized before incubating with the P2X_7_ ecto-domain antibody, and P2X_7_R expression was examined by flow cytometry and fluorescence microscopy. We found similar levels of total P2X_7_R protein in permeabilized neutrophils that were either unstimulated or LPS-primed (Fig. 2b,c). P2X_7_R was also detected on the cell surface of non-permeabilized cells (Fig. 2d,e), indicating that P2X_7_R is constitutively expressed on the cell surface of murine neutrophils.

To examine the ionotropic function of cell-surface P2X_7_R, cytosolic [Ca^2+^] bone marrow neutrophils from C57BL/6 and *P2X*_*7*_^−/−^ mice were primed with LPS and loaded with Fluo-4-AM Ca^2+^ indicator dye. Stimulation with 3 mM ATP elicited a rapid and sustained rise in intracellular [Ca^2+^] concentration (Fig. 2f). In contrast, ATP-stimulated *P2X*_*7*_^−/−^ neutrophils (Fig. 2f), and C57BL/6 neutrophils that were incubated with the highly specific P2X_7_ antagonists AZ10606120 or A438079 (Fig. 2g,h) exhibited only a transient increase in cytosolic [Ca^2+^], which rapidly decayed to baseline. This transient increase likely indicates rapid mobilization of intracellular Ca^2+^ stores by G-protein-coupled P2Y_2_ receptors, which are also activated by ATP, and is consistent with an earlier study showing that ATP elicits transient Ca^2+^ mobilization in *P2X*_*7*_^−/−^ macrophages^21^.

Quantification of the areas under the curve (AUC) for each Ca^2+^ transient showed significant reduction in the integrated increase in intracellular [Ca^2+^] in *P2X*_*7*_^−/−^ neutrophils and in C57BL/6 neutrophils treated with P2X_7_ antagonists (Fig. 2i–k).

As additional controls, neutrophils from C57BL/6 and *P2X*_*7*_^−/−^ mice were stimulated with fMLP or opsonized zymosan, and IL-1β secretion was examined. Although fMLP induced increased cytosolic Ca^2+^, there was no detectable IL-1β secretion (Supplementary Fig. 2A,B). In contrast, opsonized zymosan did induce IL-1β secretion from murine neutrophils; however, this was independent of P2X_7_R (Supplementary Fig. 2C).

Taken together, these data clearly demonstrate that murine bone marrow neutrophils express functional cell-surface P2X_7_R that respond to extracellular ATP by facilitating sustained increase in cytosolic [Ca^2+^], which is indicative of non-selective cation channels. These findings also indicate that in the absence of P2X_7_R expression or in the presence of highly selective P2X_7_ antagonists, activation of other ATP receptors, such as P2Y_2_, elicits only a transient increase in cytosolic [Ca^2+^].

### P2X_7_R-mediated IL-1β secretion by murine neutrophils

**Figure 3:**
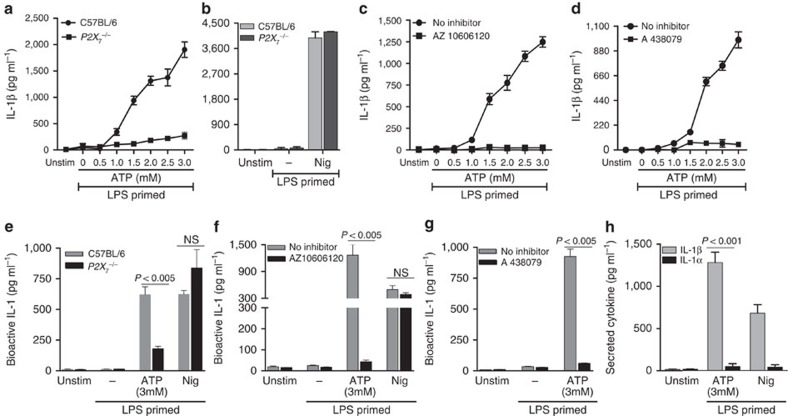
P2X_7_R-dependent IL-1β secretion by ATP-stimulated murine neutrophils. Total IL-1β secreted by bone marrow neutrophils from C57BL/6 and *P2X_7_*^−/−^ mice (**a**,**b**), or from C57BL/6 neutrophils incubated with the P2X_7_ antagonists AZ10606120 (10 μM) (**c**) or A438079 (25 μM) (**d**) following LPS priming and stimulation with ATP or nigericin measured by ELISA. (**e**–**g**). Bioactive IL-1 from the same samples detected using HEK-Blue-IL-1R reporter cells. (**h**). IL-1α and IL-1β from the C57BL/6 neutrophil supernatants stimulated 45 min with 3 mM ATP and 10 μM nigericin were quantified by ELISA. Data points are mean±s.d. of at least three replicates per treatment and are representative of three independent experiments. Using an ANOVA with Tukey *post hoc* analysis, a *P* value ≤0.05 was considered significant.

To examine the role of P2X_7_R in ATP-stimulated IL-1β secretion, LPS-primed C57BL/6 and P2X_7_^−/−^ neutrophils were stimulated with extracellular ATP or nigericin, and IL-1β secretion was quantified by enzyme-linked immunosorbent assay (ELISA). We found significantly less ATP-stimulated IL-1β in *P2X*_*7*_^−/−^ compared with C57BL/6 neutrophils (Fig. 3a), whereas there was no difference in nigericin-induced release of IL-1β (Fig. 3b). Consistent with this observation, ATP-induced IL-1β secretion by C57BL/6 neutrophils was completely inhibited in the presence of P2X_7_ antagonists AZ10606120 or A438079 (Fig. 3c,d). AZ10606120 and A438079 also inhibited IL-1β secretion induced by BzATP (Supplementary Fig. 2D).

To ascertain whether the secreted IL-1β as detected in the ELISA is bioactive, we incubated cells supernatants from ATP-stimulated neutrophils with the HEK-Blue-IL-1R reporter cell line. As shown in Fig. 3e, bioactive IL-1 was detected in LPS-primed C57BL/6 neutrophils stimulated with ATP or nigericin; however, production of biologically active IL-1 was significantly reduced in ATP-stimulated *P2X*_*7*_^−/−^ neutrophils and in C57BL/6 neutrophils incubated with P2X_7_R antagonists AZ10606120 and A438079 (Fig. 3e–g). Further, neither gene knockout nor pharmacological antagonism of P2X_7_R suppressed production of biologically active IL-1 by nigericin-stimulated neutrophils. Given the small amount of IL-1α production compared with IL-1β (Fig. 3h), the biologically active IL-1 released from the ATP-activated neutrophils appears to be primarily due to IL-1β.

Taken together, these data demonstrate that the P2X_7_R on murine neutrophils mediates production of bioactive IL-1β in response to extracellular ATP.

### Functional P2X_7_R expression on human neutrophils

**Figure 4:**
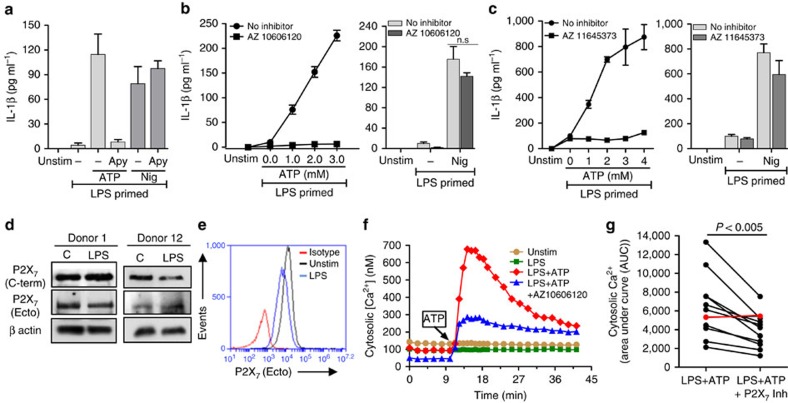
P2X_7_R expression and function on human peripheral blood neutrophils. (**a**–**c**). IL-1β measured by ELISA in the supernatant of human peripheral blood neutrophils primed with LPS (500 ng ml^−1^) for 3 h and stimulated with 4 mM ATP or 10 μM nigericin in the presence or absence of 10U ml^−1^ apyrase (**a**), or with increasing concentrations of ATP in the presence of P2X_7_ antagonists AZ10606120 (10 μM) or AZ11645373 (5 μM) (**b**,**c**). (**d**) Western blot analysis of P2X_7_R of unstimulated and LPS-primed human neutrophils from two donors that had been LPS primed for 3 h. β actin was used as a loading control. (**e**). Flow cytometry detection of P2X_7_R cell-surface expression on human neutrophils using antibody against the extracellular domain of the receptor. (**f**). Cytosolic [Ca^2+^] in LPS-primed human neutrophils after stimulation with 4 mM ATP in the presence of AZ10606120 (10 μM). (**g**). Area under the curve (AUC) for ATP induced cytosolic Ca^2+^ in neutrophils from all donors (*n*=11) in the presence or absence of P2X_7_R inhibitor (*P* values were obtained by paired *t*-tests, and a *P* value ≤0.05 was considered significant). Individual donors were for (**a**), Donor 8, (**b**), Donor 9, (**c**), Donor 7, (**d**), Donors 1 and 12, (**e**)—Donor 1, (**f**), Donor 2. Histograms or data points are mean±s.d. of at least five replicates per group and data shown are representative of three independent experiments with three different donor neutrophils (for **a**–**c**).

To examine whether ATP also elicits IL-1β secretion from human neutrophils, peripheral blood neutrophils were isolated from healthy donors, primed with LPS for 3 h, and stimulated with ATP or nigericin. As shown in Fig. 4a, IL-1β produced by ATP-stimulated human neutrophils was completely abrogated in the presence of apyrase, whereas there was no effect of apyrase on nigericin-induced IL-1β. As with murine neutrophils, millimolar concentrations of ATP were required for IL-1β release, and ATP-induced IL-1β secretion was significantly inhibited in the presence of the P2X_7_ antagonists AZ10606120 and AZ11645373 (Fig. 4b,c), indicating that ATP induces IL-1β production by human neutrophils through the P2X_7_ receptor. Human peripheral blood neutrophils constitutively express NLRP3 and ASC protein, although NLRP3 can be enhanced following TLR stimulation (Supplementary Fig. 2F).

P2X_7_ receptor expression and function in human neutrophils is controversial, with one study reporting intracellular, but not cell-surface P2X_7_ receptor expression^11^, and polymorphic P2X_7_ receptor variants reported in this gene, which have been linked to quantitative or qualitative differences in P2X_7_ receptor function in mice^22^,^23^,^24^,^25^ and in humans (Refs. 57-60). Given this heterogeneity, we assayed P2X_7_ receptor expression and function in human peripheral blood neutrophils from 11 healthy donors from multiple ethnicities. Neutrophils were primed for 3 h with LPS, and P2X_7_ protein expression was assayed by western blot and flow cytometry using antibodies against the C terminus and the extracellular domain of the receptor.

As shown for two donors, full length P2X_7_R was detected in unstimulated and LPS-primed neutrophils indicating constitutive P2X_7_R expression (Fig. 4d). Consistent with this observation, P2X_7_R surface expression was detected in non-permeabilized, unstimulated and LPS-stimulated neutrophils using the ecto-domain antibody (Fig. 4e), thereby demonstrating constitutive cell surface expression of P2X_7_ receptors on human neutrophils. P2X_7_ receptors were also constitutively expressed on neutrophils from different ethnic groups (Table 1).

To characterize ionotropic P2X_7_R function, neutrophils were incubated with LPS for 10 min to establish a stable baseline [Ca^2+^], then stimulated with 4 mM ATP in the presence of the P2X_7_ receptor antagonist AZ10606120. As shown in Fig. 4f, ATP induced a rapid increase in cytosolic [Ca^2+^], followed by a gradual decay over 30 min; however, the response was markedly attenuated in the presence of AZ10606120, indicating P2X_7_R activation. Increases in cytosolic [Ca^2+^] were detected within 5 seconds of adding ATP (Supplementary Fig. 2E).

Although neutrophils from all the donors tested expressed cell-surface P2X_7_ receptors as quantified by flow cytometry, we noted donor-specific differences in P2X_7_ receptor expression and function as indicated by both the magnitude of the ATP-induced cytosolic Ca^2+^ increases and the relative sensitivity of these responses to attenuation by the selective P2X_7_ antagonists AZ10606120 and AZ11645373 (Table 1).

ATP-induced cytosolic [Ca^2+^] transients for each individual are shown in Supplementary Fig. 3, and combined data showing the AUC for ATP-induced cytosolic [Ca^2+^]±P2X_7_R inhibitor over a 30-min period are shown in Fig. 4g. Neutrophils from 10 of the 11 individuals responded to ATP by increased cytosilic [Ca^2+^]; however, the AUC values were highly variable, ranging from 26 to 56%. Further, whereas the two P2X_7_R antagonists completely suppressed ATP-induced IL-1β release from human neutrophils, these inhibitors only partially attenuated the ATP-stimulated increases in cytosolic [Ca^2+^]. Although we did not examine this directly, partial inhibition ATP-induced increases in cytosolic [Ca^2+^] is consistent with the well-defined role of G-protein-coupled P2Y_2_ receptors in mediating ATP-triggered mobilization of IP_3_-sensitive Ca^2+^ stores in these leukocytes.

### P2X_7_R-induced K^+^ efflux

**Figure 5:**
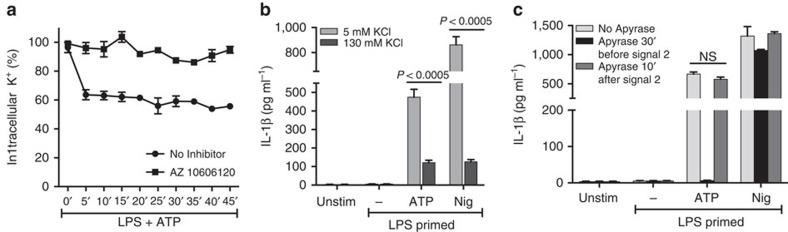
Role of K^+^ efflux in P2X_7_R-mediated IL-1β secretion by murine neutrophils. (**a**). Intracellular K^+^ content in LPS-primed C57BL/6 neutrophils stimulated with ATP (3 mM) alone and in the presence of AZ10606120 (10 μM) measured by atomic absorbance spectroscopy. (**b**). IL-1β production by LPS primed and ATP (3 mM) or nigericin (10 μM) stimulated neutrophils in the presence of 130 mM KCl or 5 mM KCl as quantified by ELISA. (**c**). IL-1β from LPS-primed C57BL/6 neutrophils in the presence of apyrase (10U ml^−1^) added either 30 min before or 10 min after stimulation with ATP or nigericin. Data points are mean±s.d. of at least four replicates per treatment and are representative of three independent experiments. A *P* value ≤0.05 was considered significant using ANOVA with Tukey *post hoc* analysis.

As non-selective cation channels, ATP-activated P2X_7_R mediates K^+^ efflux, and we and others reported that NLRP3 inflammasome activation in macrophages and dendritic cells is dependent on rapid K^+^ efflux, and can be dissociated from changes in cytosolic [Ca^2+^]^26^,^27^. As we also recently demonstrated that NLRP3 inflammasome and IL-1β secretion by pneumolysin-stimulated neutrophils requires a decrease in intracellular K^+^ concentration^28^, we examined whether there is a requirement for K^+^ efflux in ATP-induced IL-1β secretion from neutrophils. LPS-primed C57BL/6 neutrophils were stimulated with 3 mM ATP, and the intracellular K^+^ content was quantified by atomic absorbance spectrometry. Figure 5a shows a ∼30% decrease in intracellular K^+^ within 5 min of ATP stimulation, which was abrogated in presence of the P2X_7_R antagonist AZ10606120.

To determine whether this decrease in intracellular K^+^ was sufficient to drive inflammasome activation and IL-1β secretion, primed neutrophils were incubated in medium with elevated extracellular [K^+^] saline (130 mM KCl), which eliminates the outwardly directed K^+^ gradient that normally favors K^+^ efflux via gated cation channels. As shown in Fig. 5b, elimination of the K^+^ gradient in 130 mM KCl resulted in significantly lower ATP-induced IL-1β secretion compared with 5 mM isotonic KCl, indicating that P2X_7_R-driven IL-1β release from neutrophils is dependent on K^+^ efflux.

To ascertain whether the rapid decrease in intracellular K^+^ during the initial several minutes of ATP stimulation was sufficient to trigger NLRP3 inflammasome assembly and IL-1β secretion, LPS-primed neutrophils were stimulated with 3 mM ATP in the presence of apyrase, and IL-1β secretion was quantified after 45 min. ATP—induced IL-1β secretion was completely abrogated when cells were incubated with apyrase before stimulation with ATP (Fig. 5c). However, addition of apyrase 10 min after stimulation with ATP did not inhibit IL-1β secretion, indicating that only a transient activation of P2X_7_R channels is required to initiate the cascade of NLRP3 inflammasome assembly that results in proteolytic maturation and IL-1β secretion by neutrophils.

### P2X_7_R expression in *S. pneumoniae* corneal infection

**Figure 6:**
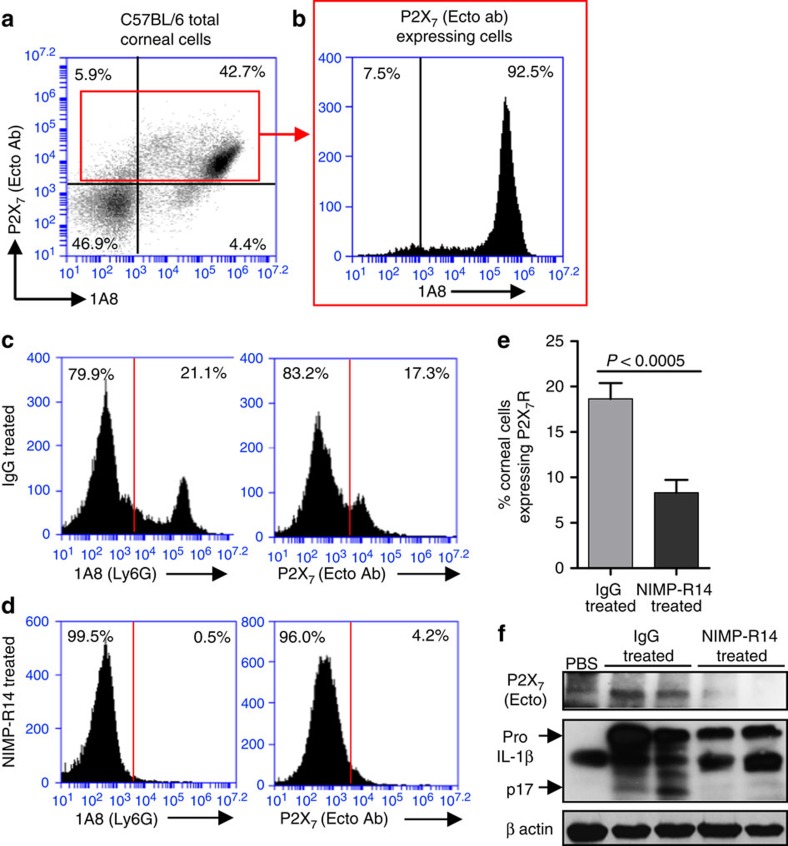
Neutrophil P2X_7_ receptor expression in *S. pneumoniae* corneal infection. P2X_7_ receptor expression on total cells (**a**), and on Ly6G+ (1A8) neutrophils (**b**) 24 h after *S. pneumoniae* corneal infection. Cells were obtained following collagenase digestion, incubated with 1A8 and antibodies against the extracellular domain of the P2X_7_ receptor and analysed by flow cytometry. (**c**–**f**) Neutrophil depletion following intraperitoneal injection of NIMP-R14 (Ly6G) or control rat IgG. (**c**,**d**) 1A8 and P2X_7_-expressing cells in the corneas 24 h post *S. pneumoniae* infection. One representative cornea is shown from each group. (**e**) Percentage and standard error of P2X_7_-receptor-expressing cells in IgG and NIMP-R14-treated mice (*n*=8 corneas). (**f**) P2X_7_ receptor, and pro- and mature IL-1β in infected corneas. Data are representative of two independent experiments with at least 5 mice per group. A *P* value ≤0.05 was considered significant using a Student's *t-*test.

Bacterial and fungal infections of the cornea are leading causes of blindness worldwide, and murine models reveal neutrophils to be prominent infiltrating cells, especially early after infection^28^,^29^,^30^,^31^,^32^. To determine whether neutrophils express P2X_7_R *in vivo*, corneas of C57BL/6 mice were infected with *S. pneumoniae*, and after 24 h, corneas were digested with collagenase, and Ly6G^+^ neutrophils and P2X_7_R^+^ cells were detected by flow cytometry. Figure 6a shows a distinct population of P2X_7_R-expressing neutrophils in the cornea, which comprise >40% of the total corneal cells. When gated on the total corneal P2X_7_^+^ population, >90% were Ly6G^+^ (Fig. 6b), indicating that neutrophils are the predominant P2X_7_R-expressing cells in the *S. pneumoniae* infected corneas.

As a second approach, neutrophils were depleted following intraperitoneal injection with NIMP-R14 antibody (anti-Ly6G) or control IgG. Corneas were then infected with *S. pneumoniae*, and P2X_7_R-expressing cells were detected by flow cytometry. As shown in Fig. 6c, IgG-treated C57BL/6 mice showed a distinct clear peak of infiltrating neutrophils (∼21%) and P2X_7_R-expressing cells (∼17%) in infected corneas. In contrast, NIMP-R14-treated mice had no infiltrating corneal neutrophils indicative of systemic depletion of neutrophils. Moreover, with depletion of neutrophils, corneal P2X_7_R-expressing cells were also significantly reduced (Fig. 6d). Figure 6e shows significantly less P2X_7_R-expressing cells in infected corneas from NIMP-R14-treated compared with control mice. Further, neither P2X_7_R nor mature IL-1β (p17) were detected in NIMP-R14-treated mice (Fig. 6f).

Taken together, these data indicate that neutrophils are the primary source of P2X_7_R and mature IL-1β *in vivo* during *S. pneumoniae* corneal infection.

### Neutrophil P2X_7_R-mediated bacterial killing *in vivo*

**Figure 7:**
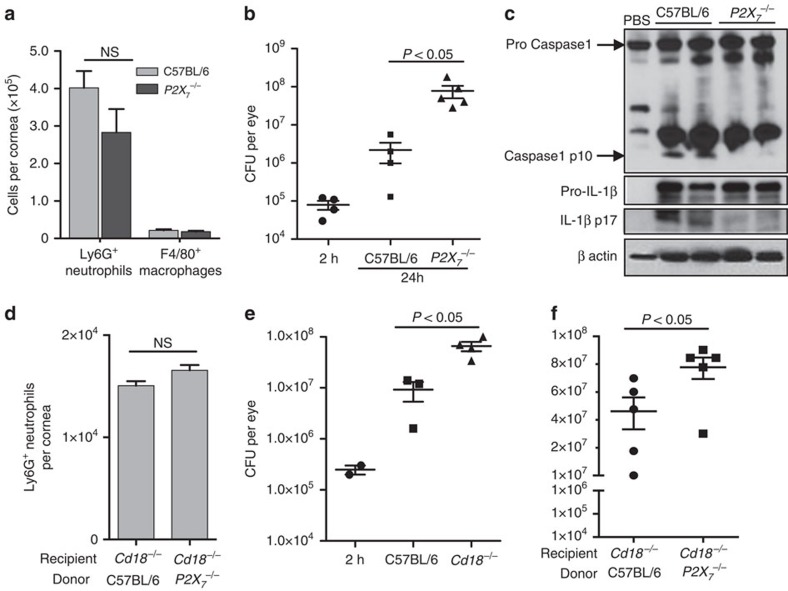
The role of neutrophil P2X_7_ receptor expression on *S. pneumoniae* corneal infection. (**a**–**c**). *S. pneumoniae* infected corneas of C57BL/6 and *P2X_7_*^−/−^ mice. Total neutrophils and macrophages quantified by flow cytometry (**a**), and bacterial CFU (**b**) 24 h post infection. (**c**). Western blot analysis of pro- and cleaved caspase-1 and IL-1β from 24 h infected corneas. (**d**–**f**). *CD18*^−/−^ mice were infected with *S. pneumoniae* and 3 h later injected intravenously with donor bone marrow neutrophils from C57BL/6 or *P2X_7_*^−/−^ mice. Total neutrophils in infected corneas of recipient *CD18*^−/−^ mice given C57BL/6 or *P2X_7_*^−/−^ neutrophils (**d**); CFU in corneas of infected C57BL/6 and *CD18*^−/−^ mice (**e**); and in infected *CD18*^−/−^ mice given donor bone marrow neutrophils from C57BL/6 or *P2X_7_*^−/−^ mice (**f**). These experiments were repeated twice with similar results. 24 h post infection mice were sacrificed and and bacterial quantified. Each data point represents one cornea and histograms are mean±s.d. of at least three corneas per group. A *P* value ≤0.05 was considered significant using a Student's *t*-test.

To assess the role of P2X_7_R in *S. pneumoniae* infection, corneas of C57BL/6 and *P2X*_*7*_^−/−^ mice were infected, and numbers of infiltrating corneal neutrophils and macrophages and bacterial colony forming units (CFU) in the eyes were quantified 24 h post infection. As shown in Fig. 7a, there was no significant difference between C57BL/6 and *P2X_7_*^−/−^ mice in the total number of infiltrating neutrophils, and there were no differences in the relatively few macrophages in the cornea at this time point. However, there were significantly more CPU in infected corneas from *P2X*_*7*_^−/−^ compared with C57BL/6 mice (Fig. 7b), indicating impaired bacterial clearance in the absence of P2X_7_R. The cleaved forms of caspase-1 and IL-1β were also not detected in corneas from infected *P2X*_*7*_^−/−^ mice (Fig. 7c), thereby demonstrating that inflammasome activation *in vivo* is driven by P2X_7_R.

To examine whether P2X_7_R expression on neutrophils is required for host defense against *S. pneumoniae*, we used a neutrophil adoptive transfer experiment as described previously^31^,^32^. *Cd18*^−/−^ mice exhibit impaired neutrophil extravasation as they are unable to bind to ICAM-1 on vascular endothelial cells^33^. Adoptive transfer of wild type or P2X_7_^−/−^ bone-marrow-derived neutrophils into the *Cd18*^−/−^ mice will therefore ascertain the specific role of neutrophil P2X_7_R expression during *S. pneumoniae* corneal infection. *Cd18*^−/−^ mice were infected with 1 × 10^5^ *S. pneumoniae* and 3 h post infection, 8 × 10^6^ C57BL/6 and *P2X*_*7*_^−/−^ bone-marrow-derived neutrophils were transferred by tail vein injection and 24 h later infected corneas were isolated for flow cytometry and quantification of CFU. The purity of donor bone marrow neutrophil preparation was estimated by FACS analysis using Ly6G antibody (Supplementary Fig. 4A). As shown in Fig. 7d, flow cytometry analysis showed no difference in the number of infiltrating neutrophils in *Cd18*^−/−^ mice with either C57BL/6 or *P2X*_*7*_^−/−^ donor cells. Figure 7e shows significantly higher bacterial CFU in the corneas of infected CD18^−/−^ mice compared with C57BL/6 mice indicative of impaired bacterial killing in absence of neutrophil infiltration in *Cd18*^−/−^ mice. Interestingly, *Cd18*^−/−^ mice that received *P2X*_*7*_^−/−^ donor neutrophils had significantly higher bacterial CFU compared with mice that received C57BL/6 donor neutrophils (Fig. 7f).

Taken together, these data indicate that the P2X_7_R expression on neutrophils is required for NLRP3 inflammaomse activation and bacterial killing in *S. pneumoniae* corneal infection.

## Discussion

Although most studies on inflammasomes and IL-1β processing have examined macrophages and dendritic cells, several reports indicate that neutrophils are also a major source of IL-1β in infectious and inflammatory diseases. Inflammasome driven caspase-1 activation by neutrophils is the major driver of IL-1β cleavage in *Salmonella typhimurium*, *Staphylococcus aureus* and *S. pneumonia* infections, and in nigericin-stimulated cells^15^,^28^,^31^,^34^. Also, inflammasome- and caspase-1-independent IL-1β secretion by neutrophils has been reported in murine models of *Pseudomonas aeruginosa* infection and rheumatoid arthritis^30^,^35^. We recently demonstrated that human and murine neutrophils express NLRP3, and that secondary activation with bacterial pneumolysin induces K^+^ efflux and formation of multiple NLRP3/ASC specks together with active caspase-1, resulting in secretion of mature IL-1β (ref. 28). The presence of multiple specks is distinct from macrophages, which except under conditions of NLRC4 gain of function mutations^36^, form single ASC specks^37^. Also, in contrast to macrophages, activation of caspase-1 in neutrophils does not lead to pyroptosis^28^,^34^.

Although neutrophils do not undergo pyroptosis, the precursor and mature forms of caspase-1 and IL-1β are secreted after ATP or nigericin stimulation. A similar mechanism occurs in human monocytes, where pro IL-1β and caspase-1 are packaged together in late endosomes or in secretory lysosomes, resulting in release of pro- and mature forms simultaneously^38^,^39^. Similarly, precursor caspase-1 and IL-1β are compartmentalized in microvesicles in ATP-stimulated human monocytes and dendritic cells, resulting in IL-1β processing before release of pro- and mature forms of the proteins^21^,^40^,^41^. It has yet to be determined if neutrophils utilize a similar non-lytic mechanism to concomitantly release pro- and mature forms of caspase-1 and IL-1β.

We reported that the exogenous pore-forming bacterial toxin pneumolysin drives K^+^ efflux-dependent NLRP3 inflammasomes activation in neutrophils^28^, and in that report and in the current study, we show that IL-1β secretion by murine neutrophils requires priming with either LPS or with heat killed *S. pneumoniae* before stimulation with pneumolysin or ATP. However, during *S. pneumoniae* corneal infection, live bacteria appear to provide both the priming and inflammasome activation signals required to generate the cleaved form of IL-1β.

Results from the current study also demonstrate that endogenously expressed ion channels trigger this pathway in murine and human neutrophils. P2X_7_R is a non-selective cation channel that mediates rapid influx of Ca^2+^ and Na^+^, and efflux of K^+^ in response to high concentrations of ATP, which results in NLRP3 activation in macrophages and dendritic cells^16^,^42^,^43^. However, several reports indicate that P2X_7_R is either not expressed on the cell surface of human neutrophils, or is present but non-functional^11^,^12^,^13^,^14^.

In the current study, we demonstrate constitutive surface expression of P2X_7_R on murine and human neutrophils, and show ATP-driven NLRP3/caspase-1 activation and cleavage and secretion of IL-1β by these cells. Our findings also provide a mechanistic context for earlier reports showing P2X_7_R transcripts in HL-60 promyelocytes, differentiated HL-60 granulocytes and human blood neutrophils^9^,^10^, and support studies showing that LPS-primed human neutrophils secrete IL-1β in response to ATP via a caspase-1-dependent pathway^44^,^45^, although these investigators did not identify the ATP-sensing receptor. Similarly, there is one report of ATP-induced IL-1β secretion by murine neutrophils^15^; however, as with human neutrophils, the ATP-sensing receptor was not defined. In addition to P2X_7_R, ATP also activates the G-protein-coupled P2Y receptors, which in neutrophils potentiates chemotaxis, superoxide production and exocytosis of primary granules^46^,^47^. However, our current findings used specific antagonists and *P2X*_*7*_^−/−^ mice to demonstrate that P2X_7_R is the critical ATP sensing receptor for NLRP3 inflammasome signalling and IL-1β secretion in murine and human neutrophils.

High concentrations of extracellular ATP generated during inflammation engage low-affinity P2X_7_R in mononuclear cells, whereas low concentrations of extracellular ATP promote either immunostimulatory or immunosuppressive effects that are mediated by activating high ATP-affinity G-protein-coupled P2Y receptors^2^,^7^. Specifically, high concentrations of ATP stimulate macrophage production of IL-1α, IL-6 and tumour necrosis factor-α, resulting in enhanced neutrophil chemotactic activity and increased adhesion to endothelial cells^48^,^49^,^50^.

Because local accumulation of extracellular ATP is critical for P2X_7_R activation, several questions regarding P2X_7_R function in neutrophils need to be considered. The requirement for millimolar levels of extracellular ATP to activate P2X_7_R is in contrast to the six other P2XR subtypes, which are gated by micromolar ATP levels. We suggest that the low ATP-affinity status of P2X_7_R minimizes inadvertent activation of maladaptive inflammatory signalling in neutrophils until they encounter damaged host cells or bacteria at sites of local tissue infection. However, given the high ATP concentration required for P2X_7_R gating, and that active ecto-ATPases are present in infected tissues, the physiological relevance has to be considered.

Junger and colleagues presented compelling evidence that neutrophils can release ATP at their leading edge when migrating in response to chemoattractant gradients^51^. In those experiments, the locally released ATP was shown to trigger G-protein-coupled P2Y_2_ receptor signalling that amplifies the rapid cytoskeletal remodelling required for efficient motility. It is therefore likely that similar autocrine and paracrine pathways exist in infected and injured tissues that may transiently and locally increase interstitial ATP levels to the millimolar levels required for activation of P2X_7_R signaling. In support of this concept, Kubes and colleagues reported activation of *in situ* P2X_7_R signaling responses by live-tissue imaging in their models of highly localized sterile injury to the liver^52^.

In the current study, we used both genetic and pharmacological approaches to demonstrate that ATP-induced IL-1β secretion is dependent on the P2X_7_R in murine and human neutrophils. First, we showed that ATP-induced IL-1β secretion is impaired in *P2X*_*7*_^−/−^ neutrophils and by highly selective P2X_7_R antagonists. Moreover, ATP stimulation of neutrophils caused rapid efflux of K^+^, which was inhibited by P2X_7_R antagonists, and was upstream of NLRP3 inflammasome activity. In addition, we showed constitutive surface expression of the P2X_7_R in murine and human neutrophils, and that in contrast to macrophages^53^, receptor expression was not increased following LPS priming. However, we found that neutrophils also constitutively expressed an intracellular pool of P2X_7_R, which raises the possibility that these receptors may be mobilized to the cell surface during inflammation.

Examination of functional P2X_7_R is complicated by the presence of both C- and N-terminal splice variants of the receptor in mice^20^,^54^. P2X_7_ knockout mice have been generated either by inserting a lacZ and a neomycin cassette into exon1 (Glaxo), or by inserting a neomycin cassette into exon 13 (Pfizer). Macrophages and T cells from Glaxo *P2X*_*7*_^−/−^ mice show enhanced IL-6 production and P2X_7_R-mediated responses, respectively^22^,^23^. This was later explained by the presence of a novel transcript variant—P2X_7_K, in the Glaxo mice, which had higher sensitivity to P2X_7_R agonist^24^. In the current study, we used bone-marrow-derived neutrophils from the Pfizer *P2X*_*7*_^−/−^ mice, and showed the presence of low levels of C-terminal transcript variants in neutrophils as detected by western blot using antibody against the extracellular domain of the receptor. Moreover, *P2X*_*7*_^−/−^ neutrophils exhibited an incomplete inhibition of IL-1β secretion, which is in agreement with reduced function of Δ*C* splice variants in the Pfizer *P2X*_*7*_^−/−^ mice^20^.

C57BL/6 mice harbour an allelic mutation (P451L) in the intracellular C-terminal domain of the P2X_7_R, whereas the non-mutated form is expressed in BALB/c mice. C57BL/6T cells expressing this Leu-451 variant exhibit a reduced rate of ATP-induced Ca^2+^ influx and attenuated apoptotic cell surface phosphatidylserine relative to BALB/c T cells expressing the Pro-451 P2X_7_R (ref. 25). Similarly, HEK cells transfected with P2X_7_-P451L complementary DNA showed reduced calcium influx compared with cells transfected with non-mutated P2X_7_ complementary DNA^25^.

However, BMDMs from C57BL/6 mice exhibit robust P2X_7_R-mediated Ca^2+^ influx responses to millimolar ATP levels that are absent in macrophages from the Pfizer *P2X*_*7*_^−/−^ mice^21^. We also compared ATP-induced processing and release of IL-1β in BMDM from C57BL/6 and BALB/c mice, and found that although BALB/c macrophages produced more IL-1β in response to lower concentrations of ATP compared with C57BL/6 macrophages, IL-1β release was equivalent at >3 mM ATP^55^. These findings are consistent with data presented in the current study, which used 3 mM ATP to stimulate C57BL/6 neutrophils to produce a robust P2X_7_-mediated Ca^2+^ influx, K^+^ efflux and processing and secretion of IL-1β.

Heterogeneity in human P2X_7_R function has been reported in healthy individuals, with marked variation in macrophage responses to ATP^56^. For example, although one report showed a correlation between ATP response and surface expression of human P2X_7_R (ref. 57), others have shown that a single-nucleotide polymorphism in the exon 13 region of human P2X_7_R results in loss of receptor function despite no loss of surface expression^58^, and other P2X_7_R polymorphisms have been identified that alter the function or the localization of the receptor^59^. Further, a single-nucleotide polymorphism (496 E→A) is present at low frequency in the Caucasian population, which results in a non-functional P2X_7_ protein when expressed as a homozygous mutation compared with partial function in heterozygous, although surface expression was normal in both groups^58^. Although P2X_7_R had no role in a murine model of Mycobacterium tuberculosis infection [REF 60 Myers], the 496 E→A loss-of-function polymorphism was found to be associated with increased susceptibility to tuberculosis^60^,^61^. Conversely, the 348A→T gain-of-function mutation is associated with increased resistance to congenital and ocular toxoplasmosis^61^,^62^.

Because extracellular ATP can stimulate increases in cytosolic [Ca^2+^] via engagement of multiple P2Y and P2X receptor subtypes, we used highly selective P2X_7_ antagonists to assess the contribution of P2X_7_R-mediated Ca^2+^ influx to the overall ATP-induced Ca^2+^ mobilization in human neutrophils. In the current study, we tested peripheral blood neutrophils from 11 healthy donors of different ethnicities and found that: (1) all donors responded to ATP; (2) 10 of 11 donors (90.1%) exhibited a P2X_7_R-dependent increase in cytosolic [Ca^2+^], with only one donor who was completely insensitive to P2X_7_ antagonism, indicating that this donor expressed a non-functional P2X_7_ variant; (3) there was considerable variation in the P2X_7_R antagonist-sensitive responses among donors; and (4) there was no apparent relationship between P2X_7_R function and ethnic origin (Table 1), although a much larger number of donors would need to be examined to determine whether there is a role for ethnicity in P2X_7_R-dependent responses with ATP. However, variation in the relative expression levels in functional P2X_7_R receptor in the neutrophils of different human subjects may in part explain discrepancies in previous studies of P2X_7_R expression in human neutrophils.

Finally, we investigated the relevance of our *in vitro* findings using a mouse model of acute *S. pneumoniae* corneal infection in which neutrophils are the major source of precursor and cleaved IL-1β (ref. 28). We demonstrate here that neutrophils are also the major P2X_7_R-expressing cells in the cornea, and showed by adoptive transfer studies that neutrophil P2X_7_R is required for IL-1β processing *in vivo*, and for optimal bacterial clearance during *S. pneumoniae* corneal infection.

In conclusion, we provide evidence of functional P2X_7_R expression in murine and human neutrophils, which mediates ATP induced NLRP3 inflammasome activation and IL-1β secretion. Since neutrophils are abundant in early stages of microbial infections, sterile inflammation, and auto-inflammatory diseases where extracellular ATP is generated, activation of P2X_7_R on these cells exacerbates inflammatory responses by mediating NLRP3 inflammasome assembly and IL-1β secretion.

## Methods

### Source of mice

C57BL/6 mice (6- to 10-week old) were from The Jackson Laboratory (Bar Harbor, ME). *Caspase1/11*^−/−^ mice were generated by Richard Flavell (Yale University, CT) as *Caspase1*^−/−^ mice and subsequently found to be deficient in caspase-11 (ref. 62). *Nlrp3*^−/−^ and *Asc*^−/−^ mice were generated by Millennium Pharmaceuticals (Cambridge, MA). *Cd18*^−/−^ mice were kindly provided by Claire Doerschuk (University of North Carolina, Chapel Hill, NC). A *P2X*_*7*_^−/−^ mouse strain was originally provided by Pfizer Global Research and Development. All the gene knockout mice were on a C57BL/6 background. Animals were housed in pathogen free conditions in microisolator cages and were treated according to institutional guidelines after approval by the Case Western Reserve University IACUC.

### Source of reagents

P2X_7_R inhibitors AZ10606120 dihydrochloride and AZ11645373, A438079 hydrochloride (Tocris Bioscience) were dissolved in DMSO and used at concentrations noted in the results. BzATP triethylammonium salt (Tocris Bioscience) was dissolved in sterile water and used at millimolar concentrations. Nigericin (Calbiochem) was used at 10 μM concentration for all *in vitro* assays. Apyrase (Sigma Aldrich) was used at 10 U ml^−1^. ATP disodium salt hydrate (Sigma Aldrich) was dissolved in water and used at micromolar concentrations. Ultrapure LPS (Adipogen) was dissolved in endotoxin free water and used at 500 ng ml^−1^ for *in vitro* assays.

### Isolation of human and mouse bone marrow neutrophils

Human neutrophils were isolated from the peripheral blood of healthy volunteers following informed consent as approved by the Institutional Review Board of University Hospitals of Cleveland. Heparinized blood was mixed with 3% dextran (Sigma Aldrich) in PBS for 20 min at room temperature (RT). The top clear layer containing leukocytes was transferred to a fresh tube and the cells were under laid with 10 ml of Ficoll Paque Plus (GE Healthcare), and centrifuged at 300*g* for 20 min. The overlying plasma was aspirated and PBMC layer was collected. The underlying neutrophil/RBC pellet was suspended in 1 × RBC lysis buffer (eBiosciences) and neutrophils were washed in sterile PBS and suspended in RPMI+L-glutamine media (Hyclone) supplemented with 2% fetal bovine serum (FBS; Mediatech). This protocol routinely yielded >97% cell purity as assessed by flow cytometry and Wrights–Giemsa stain (Sigma Aldrich).

For mouse neutrophils, total bone marrow cells were collected from tibias and femurs, and neutrophils were isolated from the total bone marrow cells by negative selection using magnetic bead-based EasySep Mouse Neutrophil Enrichment Kit (Stem Cell Technologies). Neutrophils were then washed with PBS and suspended in RPMI+L-glutamine with 1% FBS. This procedure routinely yielded >94% pure neutrophils as quantified by flow cytometry and Wright-Giemsa stain (Supplementary Fig. 1A).

### Western blot analysis

Neutrophil lysates (20–30 μg protein) were fractionated in 12% SDS–PAGE, transferred onto nitrocellulose membranes, and incubated with primary antibodies—mouse IL-1β (R&D Systems, AF-401-NA at 1:1,300), mouse Caspase-1 p10 (Santa Cruz, SC-514 at 1:200), or with antibodies targeted to the intracellular C terminus or extracellular region of P2X_7_R (Alomone Labs, catalog# APR 004 for the C terminal, and APR 008 for the ecto-domain, both at 1:200 dilution). These antibodies recognize mouse and human receptors. Loading controls were shown using antibodies to β actin (Sigma Aldrich A3854, 1:50 000). Reactivity was determined using HRP-conjugated secondary antibodies (Santa Cruz) and developed using Supersignal West Femto Maximum Sensitivity Substrate (Pierce). Images were cropped for presentation; full size images are presented in Supplementary Fig. 5.

### Flow cytometry analysis

Neutrophils were washed twice in PBS after *in vitro* stimulation. Fc receptors were blocked for 20 min at RT with anti-mouse or anti-human CD16/32 antibody (eBiosciences 16-0161-81) followed by incubation with FITC tagged anti-Ly6G (clone 1A8, Biolegend, 127613, 0.5 μg added to 1 × 10^6^ murine neutrophils in 100 μl) and anti-P2X_7_ receptor antibodies as described above. F(ab')2 donkey anti-rabbit IgG-PE (eBiosciences) was used as secondary antibody for P2X_7_. Following two washes in FACS Buffer (1% FBS in PBS), cells were fixed in 0.5% PFA for analysis by flow cytometry using an Accuri C6 Flow cytometer (Becton Dickinson).

### Intracellular Ca^2+^ assay

Human and mouse neutrophils were plated at 1 × 10^6^ per well in a 24-well plate, and incubated with LPS for 3 h. Neutrophils were then washed with PBS and transferred to basal salt solution media (130 mM NaCl, 4 mM KCl, 1.5 mM CaCl_2_, 1 mM MgCl_2_, 25 mM HEPES, 5 mM D-glucose, 1% BSA, pH 7.4) containing 1 μM Fluo-4-AM (Life Technologies) and 2.5 mM probenecid (Sigma Aldrich) for 30 min at 37 °C. Fluo-4-AM was mixed with 20% pluronic acid F-127 in a 1:1 ratio before additing to BSS media. After incubation, Fluo-4-AM containing media was aspirated, cells were washed in BSS media and then resuspended in BSS media containing 2.5 mM probenecid. Baseline readings were taken at 37 °C using a fluorescent plate reader (Synergy HT, BioTek) at 485/528 nm for 10 min, after which ATP was added to the cells and kinetic readings were obtained over 30 min. Cells were permeabilized with 1% Triton-X 100 to obtain the maximum Ca^2+^-dependent fluorescence, followed by Tris/EGTA addition to obtain Ca^2+^-independent background fluorescence.

### Fluorescence microscopy

Neutrophils were fixed with 4% PFA (Fisher) at RT for 15 min. Cells were then blocked in 10% goat serum (Vector Laboratories) in PBS for 1 h at RT, followed by staining with P2X_7_ antibody (clone Hano3, Enzo LifeScience ALX-802-027, 1:5) for 1 h at 37°C. Neutrophils were then washed three times in PBS and incubated 40 min at RT with Alexa-488 goat anti-rat IgG (Jackson Immuno Research 112-007-003, 1:300), washed three times in PBS and counter stained with DAPI before microscopy. Images were collected on a Leica DMI 6000 B inverted microscope using a × 63 objective connected to a Retiga EXi Aqua Blue camera (Q-imaging). Resulting image stacks were subjected to deconvolution using Autoquant software (Mediacybernetics). Further analysis was performed using Metamorph Imaging Software (Molecular Devices).

### Atomic absorbance spectroscopy

Neutrophils were washed in potassium-free isotonic buffer (135 mM sodium gluconate, 1.5 mM CaCl_2_, 1 mM MgCl_2_, and 25 mM HEPES). Cell pellets were then extracted in 1 ml of 10% HNO_3,_ and K^+^ content in the nitric acid extracts was quantified by atomic absorbance spectrometry (Agilent 55B AA)^28^. Triplicate samples were run for all test conditions in each experiment.

### LDH release assay

Neutrophil supernatants were collected and LDH release was quantified using CytoTox 96 Non-Radioactive Cytotoxicity Assay (Promega) according to the manufacturer's instructions. Percentage cytotoxicity was calculated based on LDH release in total cell lysates.

### ELISA

Half-well cytokine assays were performed using Duoset ELISA assay kits for murine and human IL-1β and mouse IL-1α according to manufacturer's protocol (R&D Systems).

### IL-1 Bioassay

HEK-Blue-IL-1R reporter cell line (Invivogen) was used to measure production of biologically active IL-1 (mature form of the cytokine). This reporter cell line stably expresses murine IL-1 receptor and a secreted alkaline phosphatase (SEAP) reporter gene under the control of a minimal IFN-β promoter fused to NFκB and AP-1 binding sites. The cells were cultured in 24-well plates at 2.5 × 10^5^ cells in 500 μl. They were then incubated with 5 μl supernatant from stimulated bone marrow neutrophils or with recombinant murine IL-1β standards. After 15–18 h, 20 μl aliquots of conditioned medium (either undiluted or diluted 1:5 or 1:10) from the stimulated HEK- Blue-IL-1R cells were transferred to 96-well plates containing 180 μl of QUANTI-Blue SEAP assay reagent per well. Production of blue SEAP product was measured by absorbance (620 nm) in BioTek Synergy HT plate reader, and the concentration was derived from the standard curve (known IL-1β standards) and expressed as pg per ml. As this IL-1R-based bioassay also detects IL-1α, the same samples of neutrophil-conditioned medium were assayed for murine IL-1α by ELISA (R&D).

### Murine model of *Streptococcus pneumoniae* corneal infection

*S. pneumoniae* TIGR4 (serotype IV) were grown at 5% CO_2_ in Todd-Hewitt Broth (Neogen) supplemented with 0.5% yeast extract. Bacteria were grown to mid-exponential phase (1x10^8^ CFU per ml), and 1 × 10^5^ bacteria in 2 μl sterile PBS were injected into the corneal stroma. After 24 h, corneas were dissected, and either digested in 82 U of type- I collagenase (Sigma) to recover cells for flow cytometry, homogenized in lysis buffer for western blot analysis, or whole eyes were homogenized and viable bacteria were quantified by CFU.

For flow cytometry, cell suspensions from infected corneas were passed through a 30-μm filter to remove undigested tissue, and cells were identified and quantified by flow cytometry. For CFU, whole eyes were homogenized in 1 ml sterile PBS using a Mixer Mill MM300 (Retsch). Serial log dilutions were plated on blood agar plates, incubated in a CO_2_ incubator at 37 °C for 18 h, and CFU were counted manually.

For *in vivo* neutrophil depletion, mice were injected intraperitoneally with 250 μg of the Ly6G antibody NIMP-R14 24 h before corneal infection (NIMP-R14 was generated in-house from the hybridoma). In neutrophil adoptive transfer model experiments, corneas of recipient *Cd18*^−/−^ mice were infected with *S. pneumoniae* as described above, and after 3 h, 8 × 10^6^ purified bone marrow neutrophils from donor C57BL/6 or *P2X*_*7*_^−/−^ mice were injected intravenously to the infected *Cd18*^−/−^ mice. After 24 h, corneas were collagenase digested and cells were examined by flow cytometry.

### Statistical analysis

Student's *t*-test or analysis of variance with Tukey *post hoc* analysis (Prism, Graphpad Software) were used as indicated in the figure legends. A *P* value of <0.05 was considered significant.

## Additional information

**How to cite this article:** Karmakar, M. *et al.*, Neutrophil P2X7 receptors mediate NLRP3 inflammasome-dependent IL-1ß secretion in response to ATP. 7:10555 doi: 10.1038/ncomms10555 (2016).

## Supplementary Material

Supplementary Information Supplementary Figures 1-5

## Figures and Tables

**Table 1:** P2X_7_R expression and function in peripheral blood neutrophils.

**Donor**	**Ethnicity**	**Surface P2X7R (% positive cells [MFI])**	**Ca^2+^ influx (AUC)**		**% Inhibition (AUC) of Ca^2+^ influx in presence of P2X7 inhibitor (AZ106 or AZ116)**
#1	Caucasian	Unstim—79.1% (8961)	LPS	2,591	44.2%
		LPS—68.2% (8417)	LPS+ATP	7,554	
			LPS+ATP+AZ106	4,220	
#2	Caucasian	Unstim—87.5% (8637)	LPS	3,998	43.6%
		LPS—83.8% (11,355)	LPS+ATP	13,331	
			LPS+ATP+AZ106	7,527	
#3	Caucasian	Unstim—53.5% (14.580.60)	LPS	2,323	48.6%
		LPS—52.32% (15,845.83)	LPS+ATP	10,903	
			LPS+ATP+AZ106	5,606	
#4	Caucasian	Unstim—91.9% (43,739.63)	LPS	838	28.4%
		LPS—86.9% (35,508.18)	LPS+ATP	6,640	
			LPS+ATP+AZ116	4,827	
#5	African American-Caucasian	Unstim—99.6% (30,889.34)	LPS	858	45.5%
		LPS—99.4% (24,296.22	LPS+ATP	4,460	
			LPS+ATP+AZ116	2,432	
#6	Asian	Unstim—85.6% (12,772.51)	LPS	2,307	55.9%
		LPS—86.7% (14,020.63)	LPS+ATP	7,558	
			LPS+ATP+AZ106	3,334	
#7	South east Asian	Unstim—99.2% (14,653.43)	LPS	888	33.4%
		LPS—92.9% (10,036.8)	LPS+ATP	2,782	
			LPS+ATP+AZ116	1,854	
#8	Hispanic	Unstim—72.3% (16,183.67)	LPS	4,079	26.2%
		LPS—62.9% (14,452.63)	LPS+ATP	6,120	
			LPS+ATP+AZ116	4,522	
#9	Hispanic	Unstim—95.5% (15,629.02)	LPS	1,980	33.7%
		LPS—94.3% (16,336)	LPS+ATP	4,143	
			LPS+ATP+AZ116	2,749	
#10	Caucasian	Unstim—98.1% (70,563.26)	LPS	1,353	0%
		LPS—95.9% (45,937.79)	LPS+ATP	5,325	
			LPS+ATP+AZ116	5,543	
#11	African	Unstim—93.7% (15,332.15)	LPS	1,017	42.6%
	American	LPS—75.9% (14,587.90)	LPS+ATP	2,128	
			LPS+ATP+AZ116	1,216	

AUC, area under the curve; FSC, forward scatter; SSC, side scatter.

Granulocytes from healthy donors were gated based on SSC (side scatter) and FSC (forward scatter), and 30,000 cells (unstimulated or primed with LPS for 3 h) were analyzed for surface P2X_7_ expression (extracellular antibody) within the gated population.

ATP-induced cytosolic Ca^2+^ increases and their relative sensitivity to attenuation by the selective P2X_7_ antagonists were also demonstrated. Ca^2+^ transients for each donor were quantified by measuring AUC. Attenuation of cytosolic [Ca^2+^] in presence of P2X_7_ antagonists is represented as % inhibition compared to LPS+ATP-treated cells. Individual curves for cytosolic [Ca^2+^] are shown in Supplementary Fig. 3.
