# Supplementary material for: Neutrophil P2X7 receptors mediate NLRP3 inflammasome-dependent IL-1β secretion in response to ATP
Source: Nat Commun. 2016 Feb 15;7:10555. doi: 10.1038/ncomms10555 (PMC4756306; doi:10.1038/ncomms10555)
Supplement: Supplementary Information — Supplementary Figures 1-5 [file ncomms10555-s1.pdf]

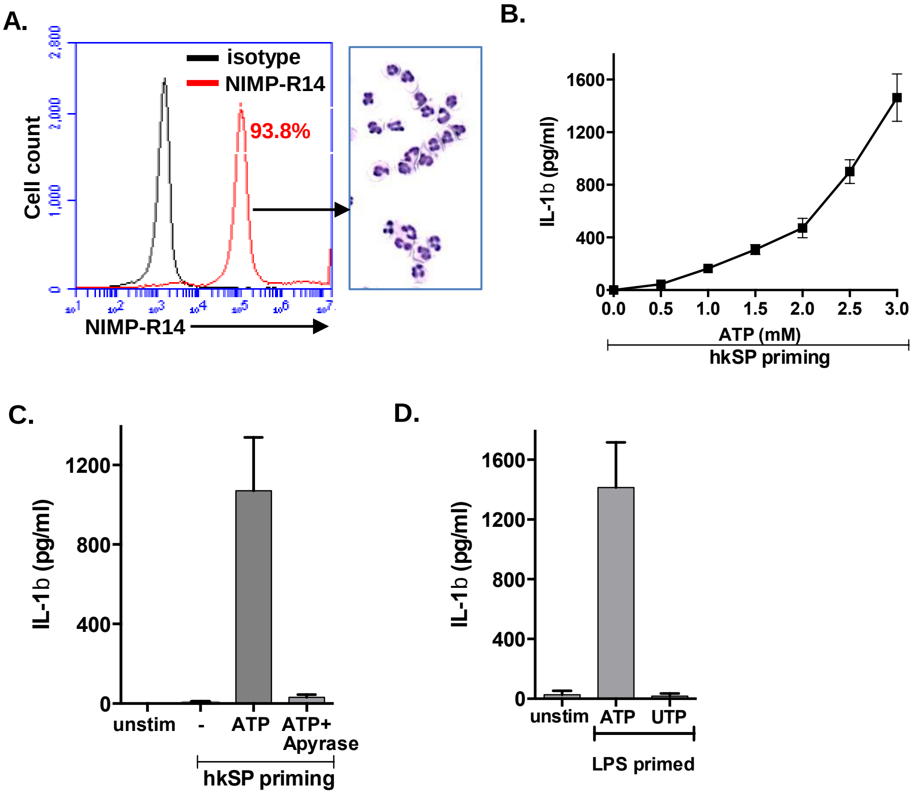

Supplementary Figure 1. IL-1β secretion by bone marrow neutrophils from ATP –stimulated C57BL/6 mice

A. Bone marrow neutrophils were purified by negative selection using magnetic beads, stained with NIMP-R14 antibody (recognizes Ly6G which is specific for neutrophils) and analyzed by FACS analysis. Wright-Giemsa staining shows the polymorphonuclear morphology of the purified neutrophils. **B**. ATP-stimulated IL-1β secretion by neutrophils primed for 3h with heat killed *Streptococcus pneumoniae* (hkSP) at a ratio of 20:1 bacteria: neutrophils. **C.** IL-1β secretion by murine neutrophils primed with hkSP for 3h and stimulated with 3mM ATP in presence or absence of 10U/ml apyrase. **D.** Bone marrow derived neutrophils from C57BL/6 mice were primed with LPS for 3h and stimulated with 3mM ATP or UTP for 45mins and IL-1β was quantified by ELISA. Each data point is mean of 4 replicates for each experimental condition.

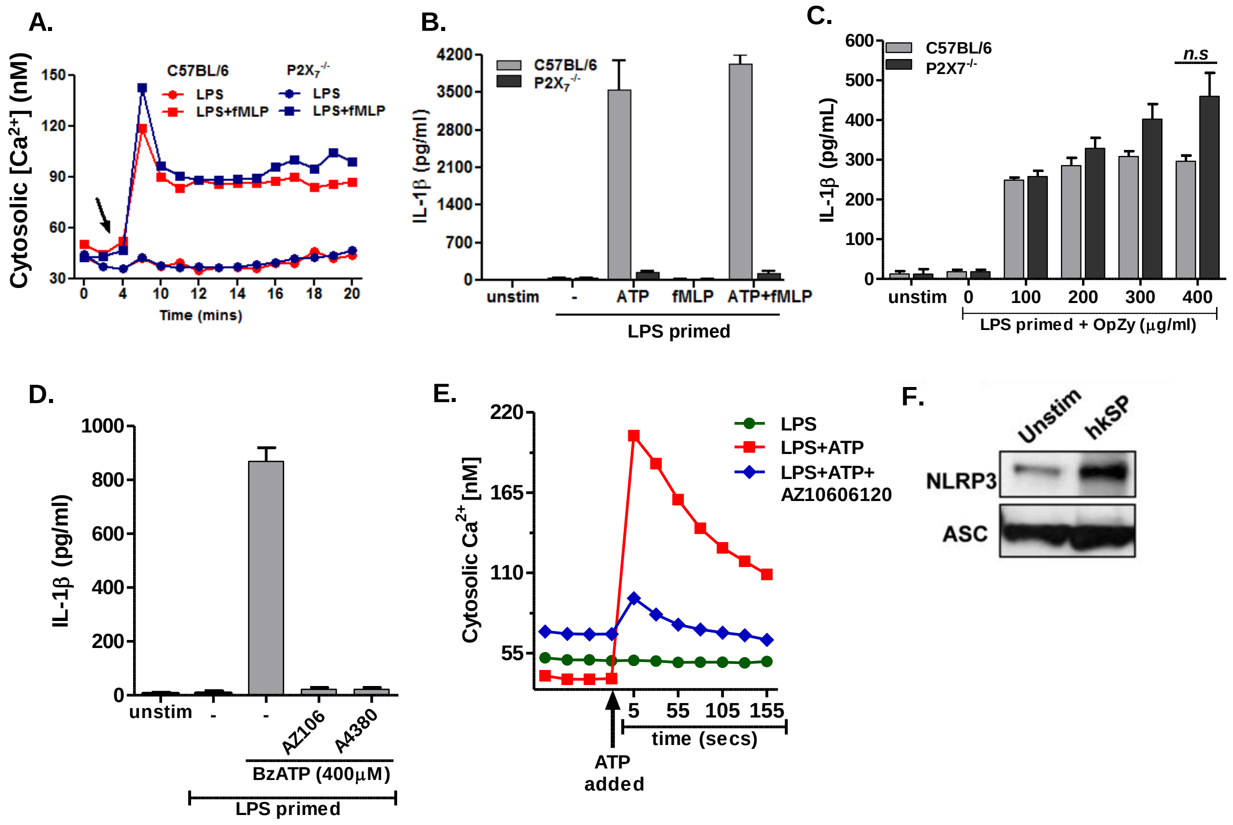

Supplementary Figure 2. IL-1β secretion by ATP –stimulated neutrophils from C57BL/6 and $P2X_7^{-/-}$ mice

A,B. Bone marrow neutrophils from C57BL/6 and $P2X_7^{-/-}$ mice were primed with LPS and stimulated with fMLP. Cytosolic [Ca^{2+}] was quantified by fluo-4-AM after stimulation fMLP **(A)** and IL-1β secretion was measured by ELISA after 45mins stimulation with ATP, fMLP and fMLP+ATP **(B)**. **C.** IL-1β secretion by bone marrow neutrophils after LPS priming followed by stimulation with opsonized zymosan for 3h. **D**. IL-1β secretion by bone marrow derived neutrophils from C57BL/6 mice were primed with 500ng/ml of LPS for 3hr and stimulated with 400μM of BzATP alone on in presence of $P2X_7$ inhibitor AZ10606120 (10μM) or A438079 (25 μM). **E**. Bone marrow neutrophils from C57BL/6 mice were primed with LPS and stimulated with ATP (3mM). Cytosolic [Ca^{2+}] was quantified by fluo-4 staining and reading was taken every 5secs (after addition of ATP) for total 3mins. **F.** NLRP3 and ASC protein in human neutrophils from a healthy volunteer after 2h stimulation with heat killed *Streptococcus pneumoniae* at a ratio of 20:1.

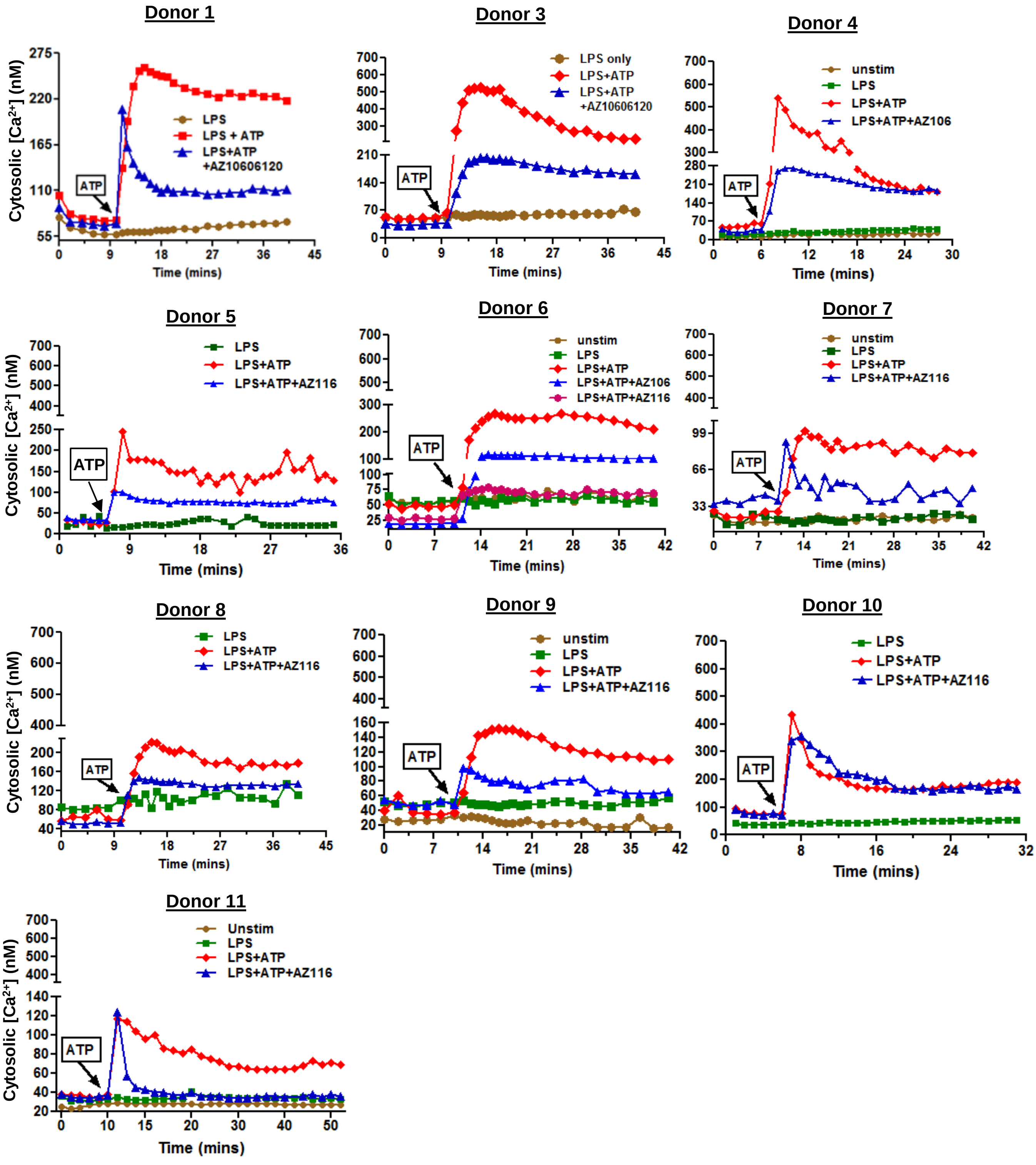

Supplementary Figure 3. $P2X_7R$ activation of human peripheral blood neutrophils from multiple donors

Peripheral blood neutrophils from healthy donors were isolated and $1x10^6$ neutrophils were plated per well. Cells were primed with LPS for 3h and stimulated with 4mM ATP in presence or absence of specific $P2X_7$ antagonists. Intracellular $[Ca^{2+}]$ was measured by fluo-4-AM. Each data point is mean of 4 replicates for each experimental condition.

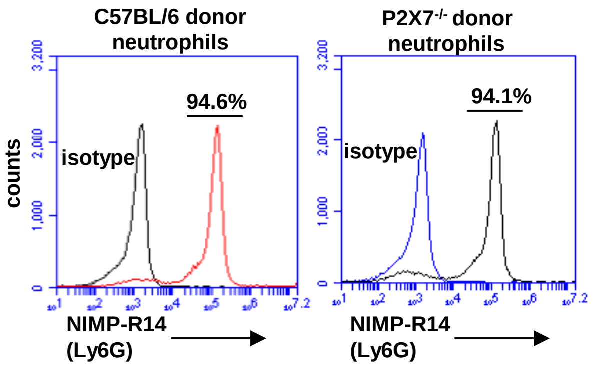

Supplementary Figure 4. Purity of donor neutrophils

Donor neutrophils for adoptive transfer experiments were isolated from C57BL/6 and $P2X_7^{-/-}$ mice and purity was checked by FACS analysis after staining with Ly6G (NIMP-R14) antibody.

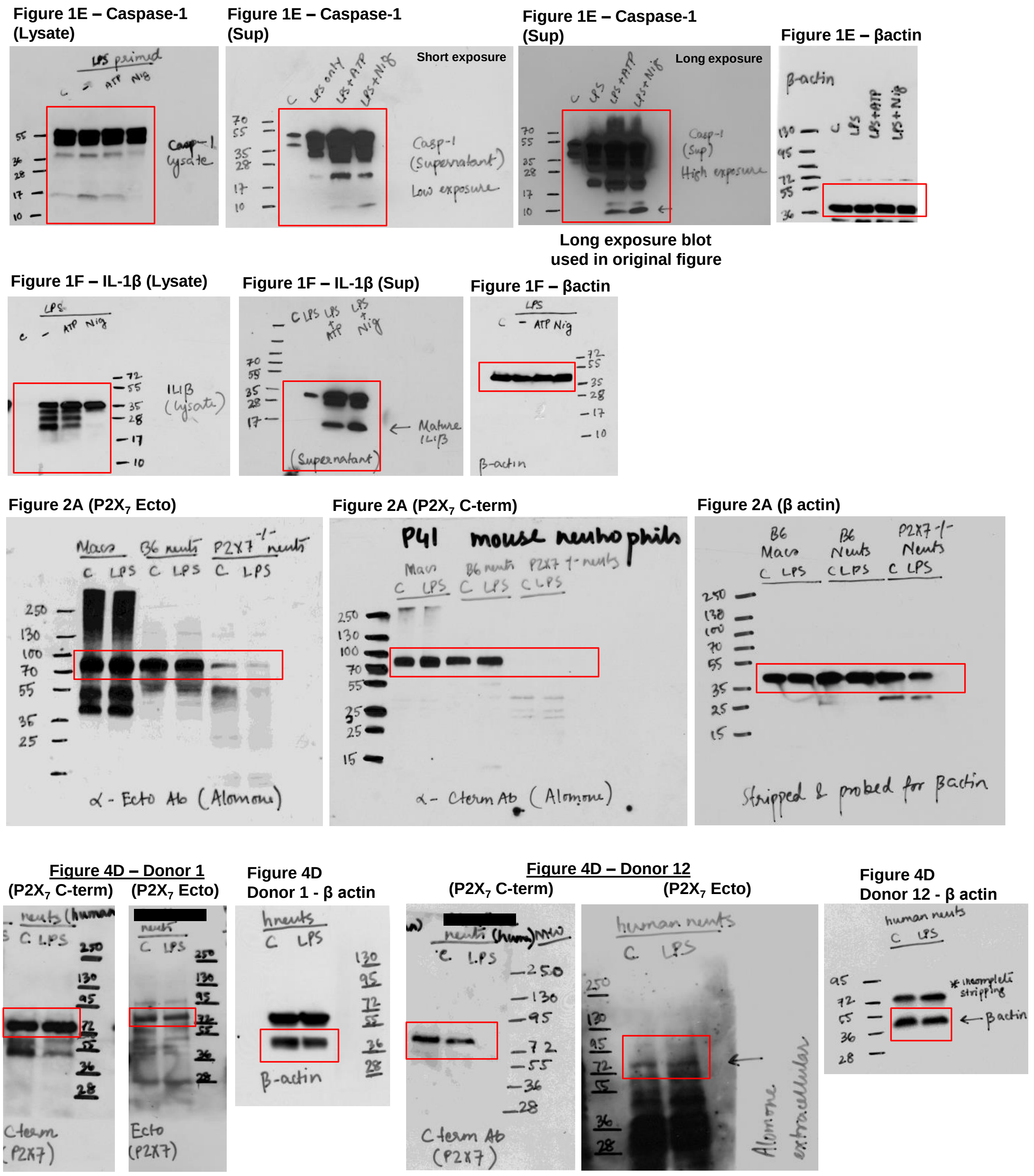

Supplementary Figure 5. Full scan of western blots

Specific protein bands that were cropped and used in the figures are marked in red boxes.

Figure 6F – Pro and mature IL-1β

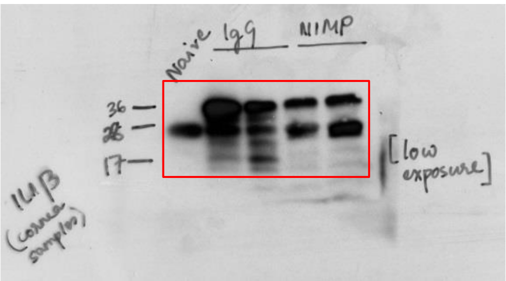

Figure 6F – P2X$_7$ (Ecto)

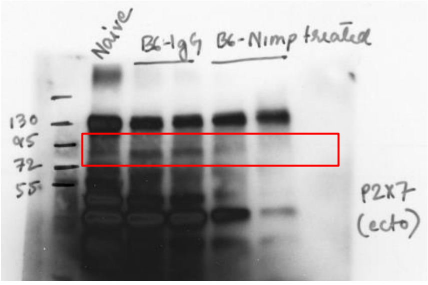

Figure 7C – Pro IL-1β

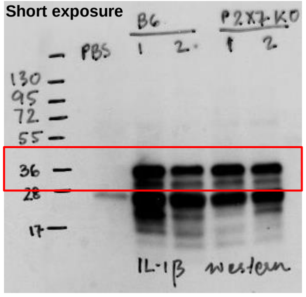

Figure 7C – Mature IL-1β

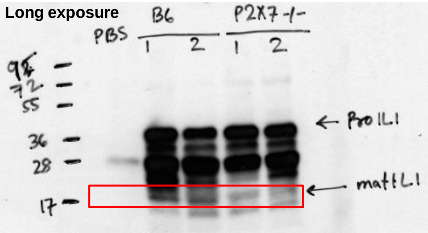

IL-1β p17 in Fig 7C was used from long exposure blot

Figure 7C – Caspase-1

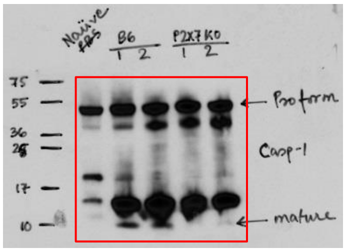

Figure 7C – β actin

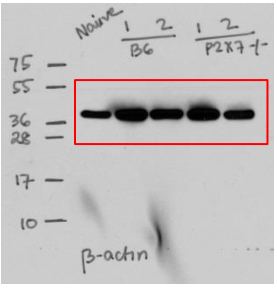

Full scan of western blot for Supplementary Figure 2F

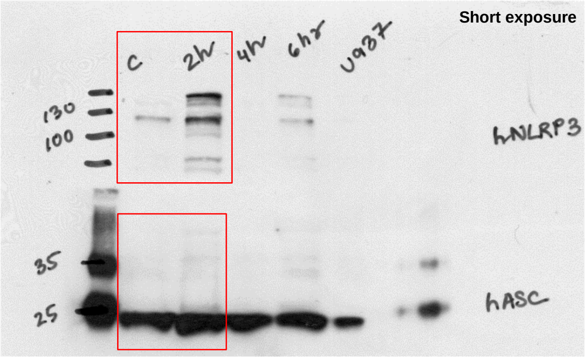

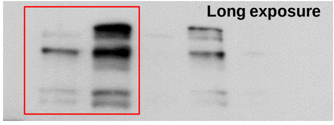

Long exposure blot used in original figure

Supplementary Figure 5. Full scan of western blots (continued)

Specific protein bands that were cropped and used in the figures are marked in red boxes.
